# Accuracy and agreement of a wearable device for heart rate and oxygen saturation monitoring in awake dogs

**DOI:** 10.1111/jsap.70162

**Published:** 2026-06-25

**Authors:** Ş. Melek, Ö. T. Orhun, M. G. Şenocak, T. Arslan

**Affiliations:** ^1^ Department of Surgery, Faculty of Veterinary Medicine Bingol University Bingöl Turkey; ^2^ Department of Surgery, Faculty of Eregli Veterinary Medicine Necmettin Erbakan University Konya Turkey; ^3^ Department of Surgery, Faculty of Veterinary Medicine Atatürk University Erzurum Turkey

## Abstract

**Objectives:**

The present study evaluated the accuracy and agreement of heart rate and peripheral oxygen saturation measurements obtained using the Owlet Smart Sock in awake dogs with low activity levels, using a veterinary patient monitor as the reference method.

**Materials and Methods:**

Clinically healthy dogs underwent simultaneous heart rate and peripheral oxygen saturation measurements with Owlet Smart Sock and a veterinary patient monitor during routine preoperative evaluation. Method comparison was performed using Pearson correlation analysis and Bland–Altman agreement analysis. Clinical equivalence was assessed using the two one‐sided tests procedure with predefined equivalence margins of ±10 bpm for heart rate and ±3% for peripheral oxygen saturation. A total of 237 paired measurements were analysed.

**Results:**

Heart rate measurements obtained with Owlet Smart Sock showed a strong positive linear relationship between the two methods (*r* = 0.934, *P* < .001). Bland–Altman analysis demonstrated a mean bias of −0.46 bpm, with narrow limits of agreement. Two one‐sided tests analysis indicated that the mean difference in heart rate measurements lay within the predefined equivalence margin (*P* < .001). In contrast, peripheral oxygen saturation measurements showed no significant correlation between devices (*r* = 0.038, *P* = .562), and Bland–Altman analysis revealed considerable variability at the individual measurement level.

**Clinical Significance:**

The Owlet Smart Sock demonstrated acceptable agreement with the reference monitor for heart rate measurement in awake dogs with low activity levels, whereas peripheral oxygen saturation measurements showed limited agreement and should be interpreted with caution in clinical practice.

## INTRODUCTION

Heart rate (HR) and peripheral oxygen saturation (SpO_2_) are among the most frequently assessed physiological parameters in veterinary clinical practice and provide essential information regarding cardiovascular and respiratory function (Pitafi et al., 2025; Salzer et al., 2022; Seyidoğlu et al., 2023). Accurate measurement of heart rate and peripheral oxygen saturation is important for assessing physiological status and supporting patient monitoring in veterinary practice. Early detection of alterations in HR and SpO_2_ may facilitate the timely identification of potential cardiorespiratory abnormalities during anaesthesia and recovery and may help reduce the risk of perioperative complications (Yanmaz et al., 2022).

Hypoxaemia may develop as a consequence of respiratory disease, cardiovascular dysfunction or circulatory impairment and, if not promptly recognised, can result in irreversible damage to vital organs (Leomiti & Ausman, 2023; Senocak et al., 2024). For this reason, reliable monitoring of HR and SpO_2_ is considered a cornerstone of effective clinical management in veterinary patients. In routine practice, veterinary monitors (VPMs) are widely used for this purpose; however, their relatively high cost, limited portability and practical constraints in certain clinical settings have encouraged the exploration of alternative or complementary monitoring approaches.

In recent years, wearable devices based on photoplethysmography (PPG) technology have gained widespread use in human medicine for non‐invasive monitoring of HR and SpO_2_ (Ali et al., 2025; Charlton et al., 2023; Liao et al., 2025). These devices offer several practical advantages, including low weight, wireless data transmission and ease of application. Nevertheless, the accuracy of PPG‐based measurements may be influenced by motion artefacts, peripheral perfusion, sensor positioning and tissue characteristics. Previous studies have reported that acceptable accuracy can be achieved under conditions of low physical activity, where motion‐related signal distortion is minimised (Dangerfield et al., 2017).

The growing availability of wearable monitoring technologies has raised interest in their potential application in veterinary medicine. However, evidence supporting their accuracy and clinical agreement in dogs remains limited. Only a small number of studies have evaluated wearable or smartwatch‐based devices for HR or SpO_2_ monitoring in canine subjects, and these have reported variable performance depending on device type, sensor location and physiological conditions (Senocak et al., 2024; Yanmaz et al., 2022).

The Owlet Smart Sock (OSS) is a sensor‐based, non‐invasive wearable device originally developed for monitoring HR and SpO_2_ in human neonates (Hasan & Negulescu, 2020). The system uses optical sensors embedded in a soft sock to detect changes in light absorption associated with pulsatile arterial blood flow. Heart rate is derived from analysis of pulsatile optical signals, whereas SpO_2_ estimation is based on the differential absorption of red and infrared light by oxygenated haemoglobin (Hasan & Negulescu, 2020; Young & Flores, 2020).

Previous studies in human medicine have reported acceptable accuracy of OSS for HR and SpO_2_ monitoring in neonatal and infant populations (Dangerfield et al., 2017; Young & Flores, 2020). To date, however, no studies have specifically evaluated the accuracy and agreement of OSS‐derived HR and SpO_2_ measurements in dogs.

The objective of the present study was to assess the accuracy and agreement of HR and SpO_2_ measurements obtained using the OSS in awake dogs with low activity levels, by comparison with reference measurements obtained using a veterinary patient monitor.

## MATERIALS AND METHODS

### Animals

The study included 20 clinically healthy mixed‐breed dogs presented to the Atatürk University Animal Hospital for elective castration. Prior to enrolment, all animals underwent a thorough physical examination and routine haematological analysis. Only dogs classified as American Society of Anesthesiologists (ASA) physical status I were included. Dogs with systemic disease or classified as ASA physical status II or higher were excluded (American Society of Anesthesiologists, 2010).

The study protocol was approved by the Atatürk University Local Ethics Committee for Animal Experiments (Approval no: 2026/02). Written informed consent was obtained from all owners.

### Clinical evaluation and measurement conditions

Heart rate and SpO_2_ measurements were obtained during routine preoperative clinical evaluation, prior to induction of general anaesthesia. No premedication or oxygen supplementation was administered during the measurement period. All measurements were performed while dogs were awake, spontaneously breathing, calm and at a low activity level. These assessments formed part of the standard pre‐anaesthetic examination aimed at evaluating anaesthetic suitability and reducing perioperative risk.

### Measurement devices and procedures

A veterinary patient monitor (VPM; Cardell 9405, Sharn Veterinary Inc., Tampa, FL, USA) was used as the reference method. Heart rate measurements were obtained *via* electrocardiography (ECG), while SpO_2_ values were recorded using the monitor's transcutaneous pulse oximetry probe positioned on the tongue.

To verify the accuracy of HR measurements obtained from the VPM and to minimise potential device‐related measurement bias, simultaneous manual auscultation using a stethoscope was performed during data collection. This served as an independent reference to confirm the reliability of the recorded HR values. Respiratory rate was assessed by visual observation of thoracic excursions as a standard non‐invasive clinical method.

For application of the Owlet Smart Sock (Owlet Baby Care Inc., Lehi, UT, USA), hair at the tibial region was clipped circumferentially using a size 40 blade to minimise optical artefacts between the sensor and skin surface. The OSS was placed directly on the tibial region without additional adhesive tape or bandaging (Fig 1). The sock‐based design of the device provided adequate fixation throughout the measurement period.

**FIG 1:**
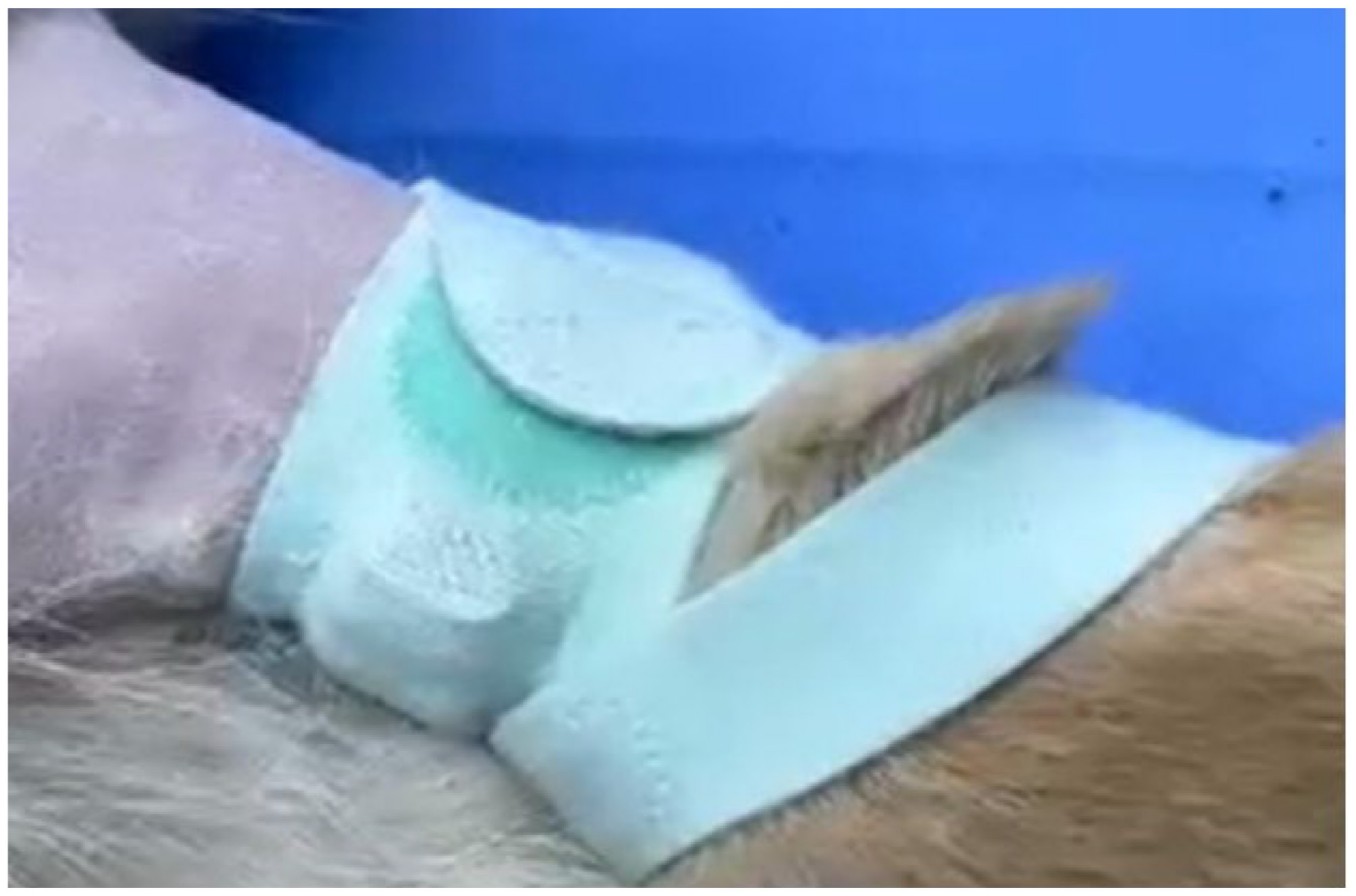
The tibial region where HR and SpO_2_ measurements were obtained using the OSS.

For the purposes of statistical analysis and graphical representation, the OSS was designated as “Device”, whereas the reference veterinary patient monitor (Cardell 9405) was designated as “Monitor”.

### Data collection

Heart rate and SpO_2_ measurements were recorded simultaneously using OSS and VPM at 1‐minute intervals over a 20‐minute period. Data collection was conducted in parallel by three investigators: one recorded OSS measurements, the second recorded VPM data, the third performed manual HR assessment and a fourth investigator evaluated respiratory rate by visual observation of thoracic movements. All measurements were subsequently transferred to an electronic spreadsheet for statistical analysis. A total of 237 paired measurements were included in the final analysis.

### Statistical analysis

Statistical analyses were structured to evaluate both association (accuracy) and agreement between measurement methods. Correlation analysis was used to assess the strength of the linear relationship between devices, whereas Bland–Altman analysis was used to evaluate agreement, including bias and limits of agreement. All statistical analyses were performed using MedCalc® software (version 20.013, MedCalc Software Ltd., Ostend, Belgium). Continuous variables were expressed as mean ± standard deviation (SD) and 95% confidence intervals (CI). Normality assumptions were assessed using visual inspection and descriptive statistics.

Agreement between measurement methods was evaluated using Bland–Altman analysis. For each comparison, mean bias and 95% limits of agreement (mean ± 1.96 SD) were calculated. Linear regression analysis was performed to assess proportional bias by examining the relationship between measurement differences and measurement magnitude.

Clinical equivalence between devices was assessed using the two one‐sided tests (TOST) procedure. Equivalence margins were predefined as ±10 bpm for HR and ±3% for SpO_2_. Equivalence was considered present when the 95% confidence interval of the mean difference lay entirely within the predefined equivalence bounds, with *P* < .05 for both one‐sided tests.

The linear relationship between devices was assessed using Pearson correlation analysis, with correlation coefficients and corresponding *P*‐values reported. Correlation strength was interpreted as follows: *r* <0.3 weak, *r* = 0.3 to 0.5 moderate, *r* = 0.5 to 0.7 strong and *r* >0.7 very strong. Statistical significance was set at *P* < .05 for all analyses.

No a priori sample size or power analysis was performed prior to the study, as the study was designed as an exploratory method comparison investigation. However, a total of 237 paired measurements were included, which is within the range commonly reported in method comparison studies. This limitation has been taken into consideration in the interpretation of the results.

## RESULTS

Dogs were between 1 and 2 years of age and weighed between 16.1 and 20.2 kg. A total of 237 paired HR and SpO_2_ measurements obtained simultaneously using the OSS and VPM were included in the analysis.

### Heart rate

Heart rate measurements obtained with OSS showed a strong positive linear relationship between the two methods (*r* = 0.934, *P* < .001; Table 1, Fig 2).

**Table 1:** Agreement analysis results of HR and SpO_2_ measurements between the OSS and the VPM (*n* = 237)

Parameter	Mean difference	95% CI (bias)	Lower limit (%)	Upper limit (%)	Correlation (*r*)	*P* (*r*)
HR	−0.46	−0.75 to −0.17	−4.91	+3.98	0.934	<.001
SpO_2_	−0.75	−0.94 to −0.57	−3.54	+2.04	0.038	.562

**FIG 2:**
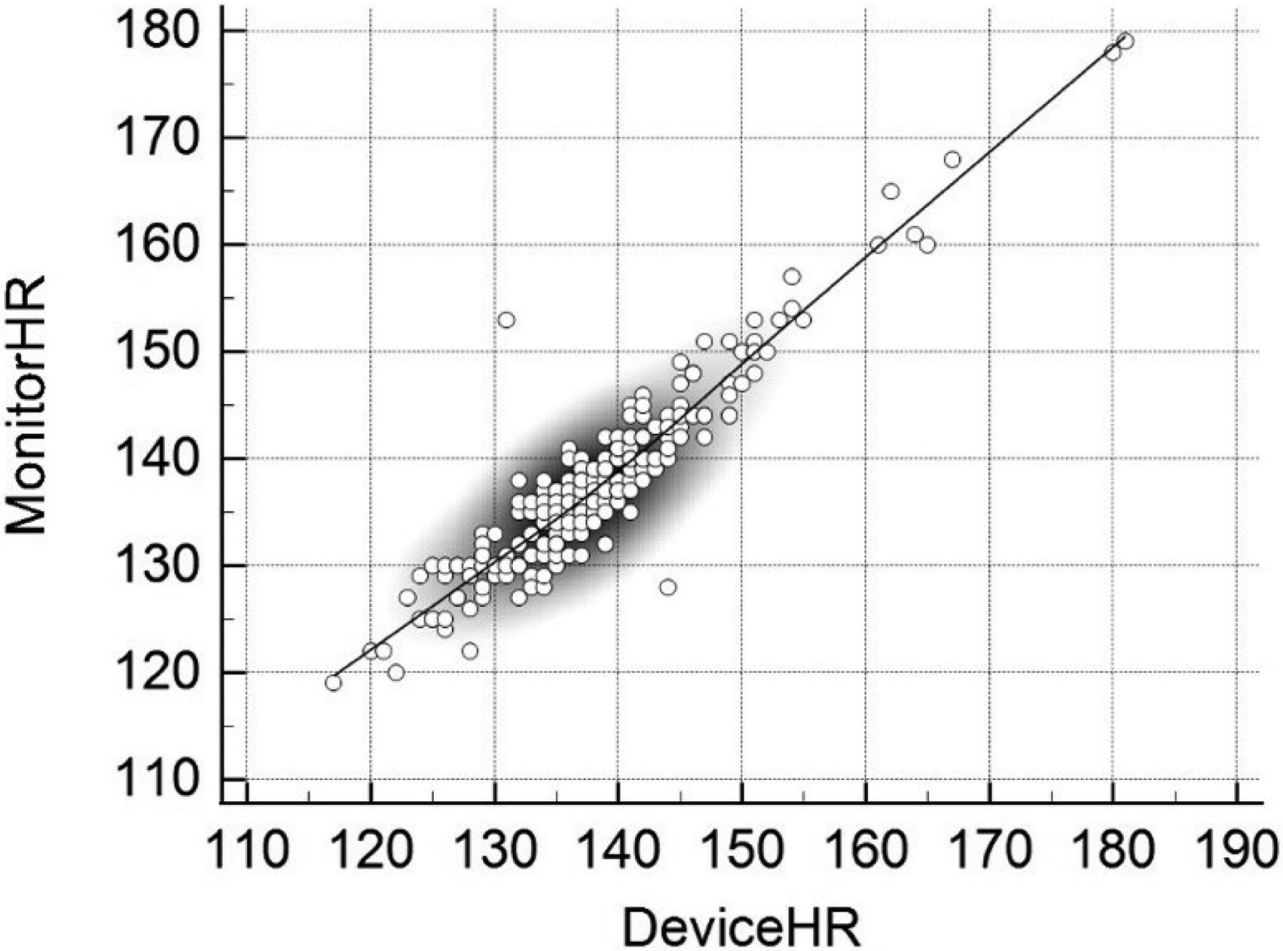
Correlation analysis of HR measurements between the OSS and the reference monitor (heat map and regression curve). The graph illustrates the relationship between HR values measured by the OSS (*x*‐axis) and the reference monitor (*y*‐axis). The density of data points is represented by a grayscale heat map. The black line represents the linear regression curve.

Bland–Altman analysis demonstrated a mean bias of −0.46 bpm, indicating a minimal tendency of OSS to underestimate HR relative to the reference monitor (Fig 3). The 95% limits of agreement ranged from −4.91 to +3.98 bpm (Table 1). Regression analysis of the measurement differences against mean HR values revealed no significant proportional bias (*P* = .688; Fig 3).

**FIG 3:**
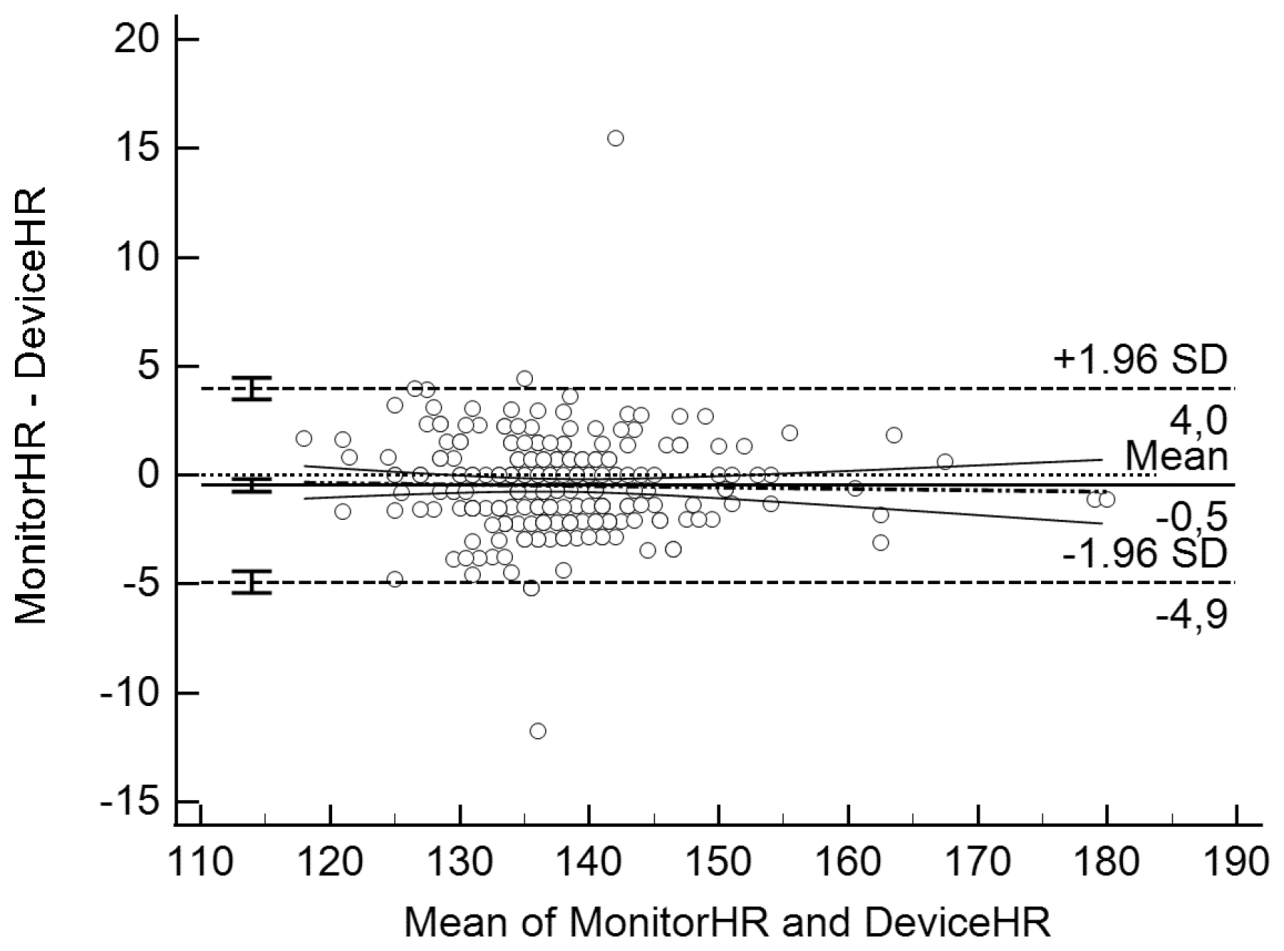
Bland–Altman analysis of HR measurements between the OSS and the reference monitor. The plot shows the distribution of the differences between HR measurements obtained from the OSS and the reference monitor against the mean of the two methods. The central horizontal line represents the mean difference (bias) between the two methods. The dashed upper and lower lines indicate the 95% limits of agreement (±1.96 SD).

Clinical equivalence analysis using the TOST procedure demonstrated that the mean difference between OSS and VPM HR measurements (−0.6 bpm; 95% CI: −1.04 to −0.25 bpm) lay entirely within the predefined equivalence margin of ±10 bpm. Both one‐sided tests were statistically significant (*P* < .001), confirming clinical equivalence between devices for HR measurement (Table 2).

**Table 2:** Two one‐sided tests (TOST) equivalence analysis of HR and SpO_2_ measurements between devices

Comparison	Mean difference	95% CI (bias)	TOST *P*‐value	Clinical equivalence
HR Monitor – device (bpm)	−0.6	−1.04 to −0.25	<.001	Equivalent
SpO_2_ Monitor – device (%)	−0.68	−0.84 to −0.51	<.001	Equivalent

Equivalence margin was set at ±10 bpm for HR and ±3% for SpO_2_

### Peripheral oxygen saturation

For SpO_2_ measurements, no significant correlation was observed between OSS and VPM values (*r* = 0.038, *P* = .562; Table 1, Fig 4).

**FIG 4:**
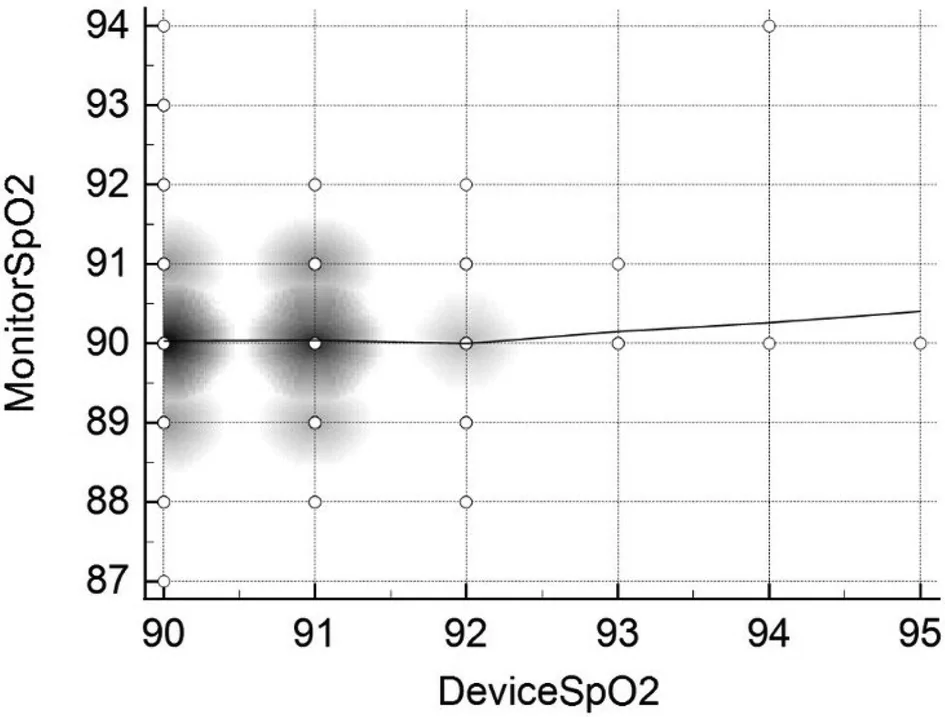
Correlation analysis of SpO_2_ measurements between the OSS and the reference monitor (heat map and regression curve). The graph illustrates the relationship between SpO_2_ values measured by the OSS (*x*‐axis) and the reference monitor (*y*‐axis). The density of data points is represented by a grayscale heat map. The black line represents the linear regression curve.

Bland–Altman analysis revealed a mean bias of −0.75%, with 95% limits of agreement ranging from −3.54% to +2.04% (Table 1; Fig 5). Regression analysis indicated a significant relationship between measurement differences and mean SpO_2_ values (*P* = .024), suggesting the presence of proportional bias (Fig 5).

**FIG 5:**
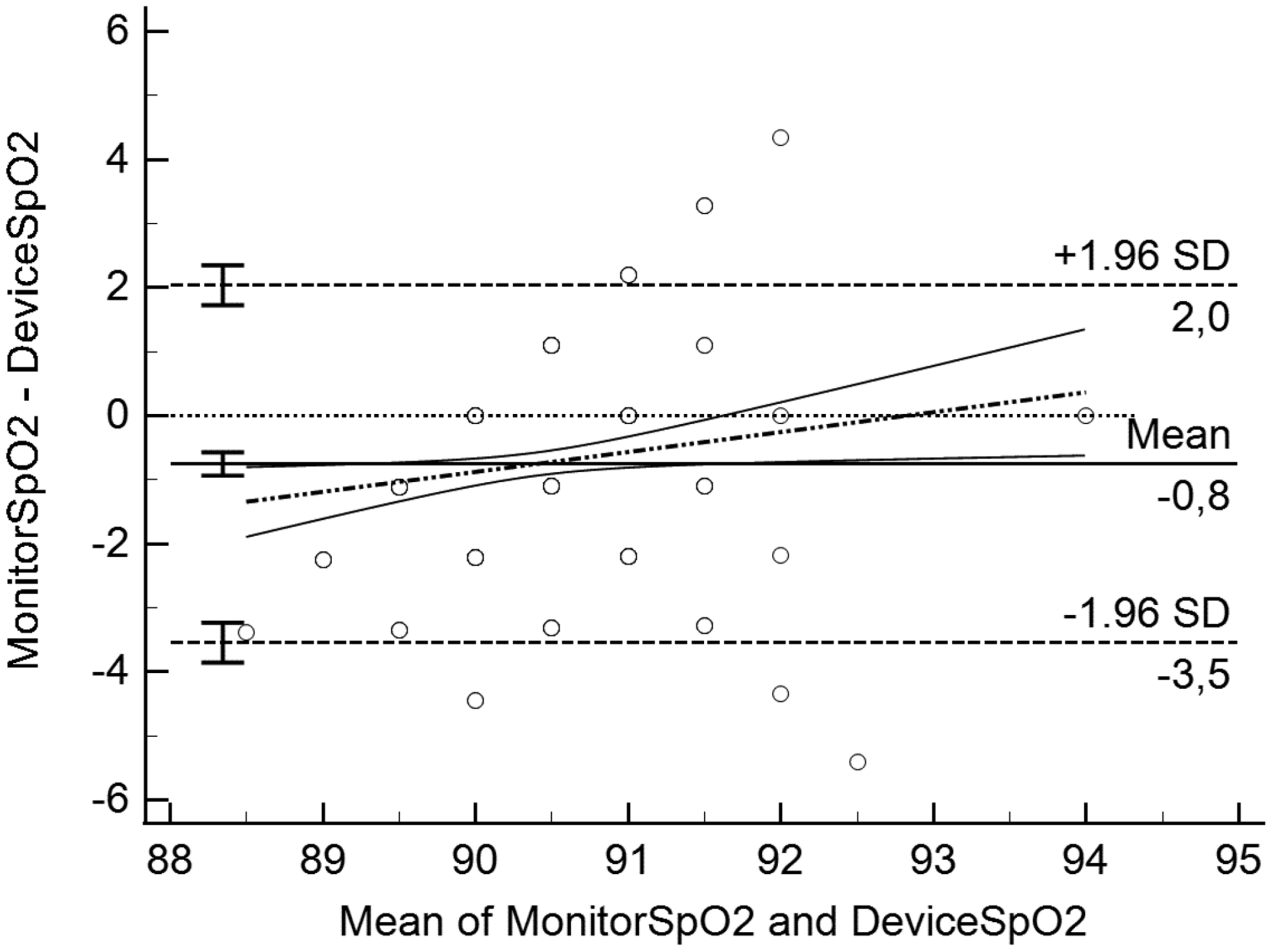
Bland–Altman analysis of SpO_2_ measurements between the OSS and the reference monitor. The graph illustrates the distribution of the differences between SpO_2_ measurements obtained from the OSS and the reference monitor plotted against the mean of the measurements from the two methods. The upper and lower dashed lines represent the 95% limits of agreement (±1.96 SD). The solid line represents the regression line indicating the relationship between measurement differences and mean SpO_2_ levels.

TOST analysis demonstrated that the mean difference between OSS and VPM SpO_2_ measurements (−0.68%; 95% CI: −0.84 to −0.51%) remained within the predefined equivalence margin of ±3%. Both one‐sided tests were statistically significant (*P* < .001), indicating that the mean difference fell within the predefined equivalence margins based on TOST analysis (Table 2).

Overall, OSS demonstrated high agreement with the reference monitor for HR measurements, as evidenced by strong correlation, minimal bias, narrow limits of agreement and confirmed clinical equivalence. In contrast, SpO_2_ measurements showed low correlation and greater variability at the individual measurement level, despite statistical equivalence of mean differences.

## DISCUSSION

In the present study, HR and SpO_2_ measurements obtained using the OSS in awake dogs with low activity levels were compared with reference values obtained using a VPM. The findings revealed that OSS showed a high level of agreement with reference monitors for HR measurements, whereas agreement was limited for SpO_2_ measurements.

HR measurements obtained with OSS showed a very strong positive linear relationship between the two methods. In addition, the mean difference was close to zero and the limits of agreement were narrow in Bland–Altman analysis, indicating clinically acceptable performance of the OSS for this parameter. These findings are consistent with previous studies reporting that PPG‐based wearable devices can provide reliable results for HR measurement under low movement conditions (Dangerfield et al., 2017; Yanmaz et al., 2022). The use of the tibial region may have contributed to measurement accuracy, as it represents an anatomical site with relatively stable peripheral perfusion. However, it should be noted that HR measurements obtained from the reference monitor were derived from ECG and manual auscultation, whereas the OSS estimates HR using PPG. Therefore, this does not represent a direct comparison of identical measurement technologies and should be considered when interpreting agreement results.

Regarding SpO_2_ measurement, no significant correlation was detected between OSS and VPM, and Bland–Altman analysis showed that measurement differences varied depending on SpO_2_ levels. These findings suggest variability between devices at the individual measurement level and the presence of proportional bias. It is known that the accuracy of SpO_2_ measurement in PPG‐based devices is significantly affected by peripheral perfusion, sensor placement, skin characteristics, and motion artefacts (Senocak et al., 2024). In dogs, the presence of hairy skin structure and extremity anatomy may also be among the factors that increase this variability. Factors such as skin pigmentation, anatomical site selection and probe positioning may also have contributed to measurement variability.

In the present study, mean SpO_2_ values obtained from both devices were lower than those typically expected in clinically healthy dogs. One possible explanation is the technical difficulty associated with pulse oximetry measurements in awake, non‐sedated animals, particularly when the tongue is used as the probe placement site. Motion artefacts, resistance to probe placement, salivation and variations in probe contact may have affected signal quality and contributed to lower SpO_2_ readings. Therefore, the possibility that the reference monitor itself may also have been affected by measurement‐related inaccuracies under these conditions cannot be excluded. This limitation should be considered when interpreting the agreement between devices, particularly for SpO_2_ measurements. Future studies incorporating arterial blood gas analysis and alternative probe placement sites such as the lip, ear, toe or prepuce may provide more reliable validation of oxygen saturation measurements in awake dogs.

In the present study, clinical equivalence analysis was also performed using the TOST method, and it was shown that the mean difference fell within the predefined equivalence margins based on TOST analysis for SpO_2_ measurements. However, it should be taken into consideration that TOST analysis provides an approach based on mean differences and does not reflect agreement at the individual measurement level, whereas the Bland–Altman method reveals the distribution at the individual measurement level and systematic bias in more detail. For this reason, even though statistical equivalence was detected for SpO_2_ measurements, OSS data should be interpreted with caution from a clinical perspective, particularly at critical oxygenation levels.

Abnormal changes in heart rate in dogs may be associated with serious cardiac conditions such as bradycardia‐tachycardia syndrome (a form of sick sinus syndrome characterised by alternating periods of abnormally low and high heart rates), sick sinus syndrome, sudden cardiac death, and transient loss of consciousness (Nakao et al., 2012; Perego et al., 2020; Santilli et al., 2021; Ware et al., 2021). In addition, early changes in cardiorespiratory parameters may also be observed in respiratory‐related pathologies such as pleural effusion (Benko et al., 2021; Pachtinger, 2013). The common feature of these clinical conditions is that findings may be transient or non‐specific in the early period and may not be detected during routine examination. Therefore, the ability to monitor HR non‐invasively and continuously may provide potential benefit as a supportive tool for clinical evaluation, especially in dogs with low activity levels. In the present study, the high level of agreement observed between OSS and VPM for HR measurements suggests that such wearable devices may be considered as an auxiliary monitoring method for the early detection of cardiac rhythm disorders.

The present study has some limitations. First, an a priori sample size or power analysis was not performed, which represents a methodological limitation. Although the number of paired measurements was relatively high, the absence of formal sample size calculation may affect the robustness of statistical inferences. In addition, OSS measurements were limited to a specific duration, and long‐term use performance was not evaluated. The high level of individual variation in SpO_2_ measurements indicates that the device may not be appropriate to be used alone in the clinical decision‐making process for this parameter. Another limitation is the use of a pulse oximeter as the reference method without validation against an invasive gold standard such as arterial blood gas analysis. Furthermore, no information was available regarding compliance of the device with established medical device standardisation frameworks, which limits direct comparison with clinically validated monitoring systems. Future studies including larger populations with different breeds, age groups, and clinical conditions are warranted.

Overall, OSS showed a high level of agreement with reference monitors for HR measurements and provided clinically acceptable accuracy. In contrast, significant variability was detected between OSS and reference monitors at the individual measurement level for SpO_2_ measurements, and systematic deviations were observed in Bland–Altman analysis. Although it was shown that mean differences remained within predefined limits in clinical equivalence analysis, it is understood that SpO_2_ measurements should be interpreted with caution, particularly at clinically critical oxygenation levels. When these results are evaluated together, it is concluded that OSS may be used as a complementary tool in veterinary clinical practice for monitoring HR in dogs using a non‐invasive and easily applicable method; however, SpO_2_ measurements should not be accepted as a sole reference in the clinical decision‐making process. The portable, wireless, and user‐friendly structure of OSS may contribute to supportive monitoring of cardiorespiratory status, particularly during the preoperative clinical evaluation process. There is a need for larger‐sample and long‐term studies involving different breeds and age groups. Such studies will more clearly reveal the potential areas of use and limitations of OSS in veterinary medicine.

In conclusion, the OSS demonstrated acceptable agreement with the reference monitor for heart rate measurement under low activity conditions in dogs. However, variability in SpO_2_ measurements and methodological limitations indicate that oxygen saturation values should be interpreted with caution. Further studies using gold standard reference methods and larger populations are required.

## Author contributions

**Ş. Melek:** Conceptualization; methodology; supervision; writing – review and editing; writing – original draft; investigation. **M. G. Şenocak:** Methodology; formal analysis. **Ö. T. Orhun:** Conceptualization; methodology; data curation. **T. Arslan:** Data curation.

## Conflict of interest

The authors declare no competing interests.

## Funding

This study was provided by Türkiye Bilimsel ve Teknolojik Araştırma Kurumu (TÜBİTAK).

## Data Availability

The article data are available from the corresponding author upon request.
